# 

**Published:** 1887-08

**Authors:** 


					﻿io cts. a copy.*
" AUGUSTU-8S7.
$1.00 per year.
HALES
heaith
ESTABLISHED 18^4
U°i- 34
oVe.- 8.
OFFICE: 206 BROADWAY, EVENING POST BUILDING, Room 11. N. Y.
Entered as Second-class Matter at the Post-Office, New York.
				

